# Kumaraswamy inverse Gompertz distribution: Properties and engineering applications to complete, type-II right censored and upper record data

**DOI:** 10.1371/journal.pone.0241970

**Published:** 2020-12-03

**Authors:** M. El-Morshedy, Adel A. El-Faheem, M. El-Dawoody

**Affiliations:** 1 Department of Mathematics, College of Science and Humanities in Al-Kharj, Prince Sattam Bin Abdulaziz University, Al-Kharj, Saudi Arabia; 2 Department of Mathematics, Faculty of Science, Mansoura University, Mansoura, Egypt; 3 Department of Mathematics, Faculty of Science, Aswan University, Aswan, Egypt; Tongii University, CHINA

## Abstract

This article proposes and studies a new three-parameter generalized model of the inverse Gompertz distribution, in the so-called Kumaraswamy inverse Gompertz distribution. The main advantage of the new model is that it has "an upside down bathtub-shaped curve hazard rate function" depending upon the shape parameters. Several of its statistical and mathematical properties including quantiles, median, mode, moments, probability weighted moment, entropy function, skewness and kurtosis are derived. Moreover, the reliability and hazard rate functions, mean time to failure, mean residual and inactive lifetimes are also concluded. The maximum likelihood approach is done here to estimate the new model parameters. A simulation study is conducted to examine the performance of the estimators of this model. Finally, the usefulness of the proposed distribution is illustrated with different engineering applications to complete, type-II right censored, and upper record data and it is found that this model is more flexible when it is compared to well-known models in the statistical literature.

## 1. Introduction

The two-parameter Gompertz (G) distribution was offered by [1] and it can be displayed as an extension of the exponential distribution. It has an influential role in survival analysis for forming adequate actuarial and human mortality tables. Also, it is a beneficial model for survival distributions characterized by increasing hazard rate and also to describe the distribution of adult life spans by demographers and actuaries; see [2]. Several authors have contributed to the studies that accentuate the statistical characterization and methodology of the G distribution; like [3–10].

The inverse distributions were introduced in the modeling literature in demography, biological and actuarial surveys; look in [10–15]. The inverse Gompertz (IG) distribution was proposed by [16] and it was introduced as a lifetime model. Suppose that *X* is a random variable that has an IG distribution whose shape and scale parameters are *λ* > 0 and *β* > 0, respectively. Then, the cumulative distribution function (CDF) of *X* takes the formula

$$G(x)=e^{-\frac{\lambda }{\beta }(e^{\frac{\beta }{x}}-1)};\;x>0;\;\lambda ,\;\beta >0.$$ (1)

Also, the probability density function (PDF) of *X* takes the formula

$$g(x)=\frac{\lambda }{x^{2}}e^{\frac{\beta }{x}}{\;e}^{-\frac{\lambda }{\beta }(e^{\frac{\beta }{x}}-1)};\;x>0;\;\lambda ,\;\beta >0.$$ (2)

[17] presented a new lifetime model having only one parameter and it is called A distribution which is characterized by an upside-down bathtub shaped hazard function. The random variable Z has an A distribution with scale parameter *β* > 0, if the CDF of Z takes the form

$$F(z)=e^{-\frac{1}{\beta }(e^{\frac{\beta }{z}}-1)};\;z>0;\beta >0.$$ (3)

The PDF of Z takes the form

$$f(z)=\frac{1}{z^{2}}e^{\frac{\beta }{z}}{\;e}^{-\frac{1}{\beta }(e^{\frac{\beta }{z}}-1)};\;z>0;\;\beta >0.$$ (4)

Setting *λ* = 1 in Eqs (1) and (2), we deduce the CDF and PDF of A distribution with the parameter *β*. That is, the A distribution is a particular case of IG distribution. In many workable circumstances, the classical distributions don’t give a sufficient fit to actual data. Therefore, various generators are proposed to produce a new models; see [18–28]. The Kumaraswamy-G (K-G) family is one of the essential generators that have an increased interest after the persuasive debate on the pitfalls of the beta-G family suggested by [29]. Cordeiro and de Castro (2011) clarified the CDF of the two-parameter K-G that takes the formula

$$F(x)=1-{\left\{1-{G(x)}^{\theta }\right\}}^{\gamma }.$$ (5)

The corresponding PDF to Eq (5) will be

$$f(x)=\gamma \theta {g(x)G(x)}^{\theta -1}{\left\{1-{G(x)}^{\theta }\right\}}^{\gamma -1},$$ (6)

where, *g*(*x*) and *G*(*x*) are the PDF and CDF of a baseline random variable *X*. Also, *θ* > 0 and *γ* > 0 are two extra shape parameters.

The principal suggest in our paper is a generalization of the IG distribution called the Kumaraswamy inverse Gompertz distribution, abbreviated KuIG, depending on the Eqs (5) and (6). The failure rate function of the new model takes the form of "an upside down bathtub-shaped". Another important characteristic of the KuIG is that it suitable for testing the goodness of fit of some special sub-models, such as the IG and A distributions. The article is distributed as follows: Section 2 introduces the CDF and the corresponding PDF of the KuIG. Section 3 presents several fundamental statistical properties. Some essential functions used in reliability analysis are introduced in Section 4. The maximum likelihood approach is mentioned in Section 5 to appreciate the parameters of the KuIG. In Sections 6 and 7, we will obtain the maximum likelihood estimators for type-II right censored and upper record data, respectively. The performance of the KuIG estimators is appreciated in Section 8 using a simulation study. We will analyze five actual data sets are three complete, one type-II right censored, and one upper record data in Section 9 and the results are compared with different known distributions. Finally, in Section 10, a conclusion for the obtained results is presented.

## 2. Kumaraswamy inverse Gompertz distribution

### 2.1. Specifications of KuIG

The non-negative random variable *X* is said to have the KuIG with the vector of parameters **Ω** = (*α*, *β*, *γ*), say *X* ∼ KuIG(**Ω**), if its CDF is given by the formula

$$F(x)=1-{\left\{1-e^{-\frac{\alpha }{\beta }(e^{\frac{\beta }{x}}-1)}\right\}}^{\gamma };\;x>0;\;\alpha ,\;\beta ,\;\gamma >0,$$ (7)

where, *α* = *λθ*. We can easily see that, if we substitute the CDF of A distribution, instead of the CDF of IG in Eq (5), we will obtain the same result. So, the proposed model can be named KuIG or KuA. The PDF of the KuIG will be

$$f(x)=\frac{\alpha \gamma }{x^{2}}e^{\frac{\beta }{x}}{e^{-\frac{\alpha }{\beta }(e^{\frac{\beta }{x}}-1)}\{1-e^{-\frac{\alpha }{\beta }(e^{\frac{\beta }{x}}-1)}\}}^{\gamma -1};\;x>0;\;\alpha ,\;\beta ,\;\gamma >0.$$ (8)

**Fig 1:**
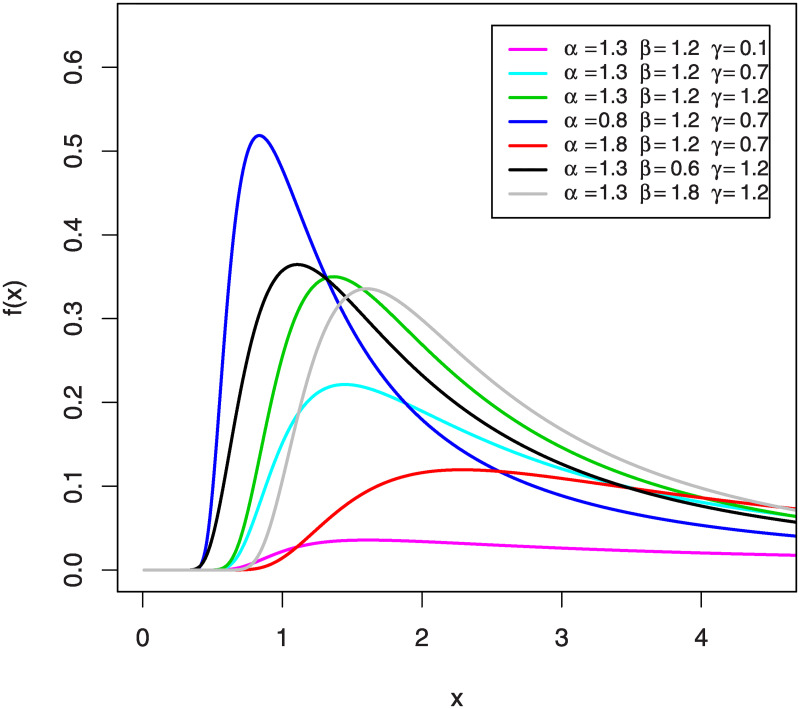
The graphs of the PDF for KuIG.

The two parameters *α* and *γ* are the shape parameters and *β* is the scale parameter. Setting *γ* = 1 in the Eqs (7) and (8), we will get the CDF and PDF of the IG with the two parameters *α* and *β*, respectively. Moreover, if we put *α* = *γ* = 1 in the above two equations, we will obtain the CDF and PDF of A distribution with the parameter *β*. This confirms the fact that the IG and A distributions are particular cases of our proposed KuIG. Fig 1 shows the graphical behavior of the PDF for KuIG with different values of *α*, *β* and *γ*.

## 3. Statistical characteristics of KuIG

### 3.1. The quantiles and median

An explicit formula for the quantile and the median of KuIG are derived in this subsection. The quantile *x*_*q*_ of the KuIG is given as follows

$$x_{q}=\frac{\beta }{ln\{1-\frac{\beta }{\alpha }\;ln(1-{\left(1-q\right)}^{\frac{1}{\gamma }})\}};0<q<1$$ (9)

The median of KuIG is found by putting $q=\frac{1}{2}$ in Eq (9) as follows

$$Med(X)=\frac{\beta }{ln\{1-\frac{\beta }{\alpha }\;ln(1-{\left(\frac{1}{2}\right)}^{\frac{1}{\gamma }})\}}$$ (10)

### 3.2. The mode

The mode of KuIG is obtained by solving the equation below with respect to *x*.

$$\alpha e^{\frac{\beta }{x}}(1-\frac{\gamma -1}{e^{\frac{\alpha }{\beta }(e^{\frac{\beta }{x}}-1)}-1})-2x-\beta =0.$$ (11)

This equation has no explicit solution in *x*. So, some numerical methods are used to solve it.

### 3.3. The *r*^*th*^ moment

If *X* ∼ KuIG(**Ω**), then the *r*^*th*^ moment of *X* is found using

$$\mu^{\left(r\right)}=E(x^{r})=\int_{0}^{\infty }x^{r}f(x)dx.$$ (12)

By substituting from Eq (8) in Eq (12), we get the *r*^*th*^ moment as follows

μ(r)=∑k=0γ-1∑i=0∞∑j=0i∑m=0∞(γ-1k)(-1)i+j+k+1(k+1)i(1+i)mαi+1βr-i-1jr-m-1γΓ(m-r+1)j!m!(i-j)!. (13)

### 3.4. Moment generating function

The moment generating function of KuIG, say *M*_*X*_(*t*), is found using

$$M_{X}(t)=E(e^{tx})=\sum_{r=0}^{\infty }\frac{t^{r}}{r!}\int_{0}^{\infty }x^{r}f(x;\Omega )dx=\sum_{r=0}^{\infty }\frac{t^{r}}{r!}\mu^{\left(r\right)}.$$ (14)

Substituting from Eq (13) into Eq (14), we obtain

MX(t)=∑r=0∞∑k=0γ-1∑i=0∞∑j=0i∑m=0∞(γ-1k)(-1)i+j+k+1(k+1)i(1+i)mαi+1βr-i-1jr-m-1γtrΓ(m-r+1)r!j!m!(i-j)!. (15)

### 3.5. The probability weighted moment

The probability weighted moment (PWM) was initially offered and introduced by [30]. The PWM of *X* with CDF *F*(*x*), say *ξ*_*s*,*r*_, is assigned by

$$\xi_{s,\;r}=E(x^{s}F^{r}(x))=\int_{0}^{\infty }x^{s}F^{r}(x)f(x)dx.$$ (16)

If *X* ∼ KuIG(**Ω**), then the PWM *ξ*_*s*,*r*_ of *X* is given by the formula

ξs,r=∑l=0r∑k=0γ(l+1)-1∑i=0∞∑j=0i∑m=0∞(rl)(γ(l+1)-1k)(-1)i+j+k+l+1(k+1)i(1+i)mαi+1βs-i-1js-m-1γΓ(m-s+1)j!m!(i-j)!. (17)

### 3.6. The entropy function and *ρ*–entropy

Entropy performs a pivotal role in engineering, information theory, computer science and probability theory. It can be used as a measure of dispersion for the uncertainty associated with a random variable *X*; see [31]. The Rényi entropy of *X* with PDF (*x*), say *I*_*ρ*_(*X*), is expressed by

$$I_{\rho }(X)=\frac{1}{1-\rho }log\int_{0}^{\infty }f^{\rho }(x)dx,\;\rho \in ]0,\;\infty [-\{1\}.$$ (18)

If *X* ∼ KuIG(**Ω**), then *I*_*ρ*_(*X*) is given by the formula

Iρ(X)=11-ρlog[∑k=0ρ(γ-1)∑i=0∞∑j=0i∑m=0∞(ρ(γ-1)k)(-1)i+j+k+1(ρ+k)i(ρ+i)mαρ+iβ-2ρ-i+1j-2ρ-m+1γρΓ(2ρ+m-1)j!m!(i-j)!]. (19)

The *ρ*–entropy of *X*, say *H*_*ρ*_(*X*), is found by

$$H_{\rho }(X)=\frac{1}{1-\rho }log[1-(1-\rho )I_{\rho }(X)].$$ (20)

### 3.7. Skewness and kurtosis

The effect of the shape parameters *α* and *γ* on skewness (*S*_*k*_) and kurtosis (*K*_*u*_) is investigated using the quantiles of KuIG given in Eq (9) [32] suggested skewness using quartiles called the Bowley skewness which is defined as follows

$$S_{k}=\frac{q(0.75)+q(0.25)-2q(0.5)}{q(0.75)-q(0.25)}.$$ (21)

**Fig 2:**
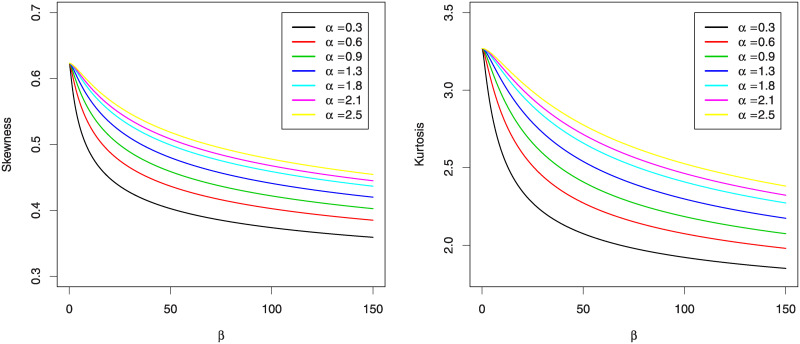
The graphs of the *S*_*k*_ (left panel) and the graphs of the *K*_*u*_ (right panel) for KuIG distribution.

Also [33], proposed kurtosis based on octiles called the Moors kurtosis which is defined as

$$K_{u}=\frac{q(0.375)+q(0.875)-q(0.625)-q(0.125)}{q(0.75)-q(0.25)},$$ (22)

where, q(.) is the quantile function. Fig 2 gives the graphs of *S*_*k*_ and *K*_*u*_ for some values of *α* when *γ* = 0.6. This figure reveals that both measures *S*_*k*_ and *K*_*u*_ decreases when *β* increases for fixed *α*.

## 4. Reliability analysis

### 4.1. The survival and failure rate functions

The survival (reliability) function of *X* ∼ KuIG(**Ω**) is found by the formula

$$R(x)={\left\{1-e^{-\frac{\alpha }{\beta }(e^{\frac{\beta }{x}}-1)}\right)}^{\gamma };\;x>0;\;\alpha ,\;\beta ,\;\gamma >0.$$ (23)

The failure (hazard) rate function (HRF) of *X* is found by the formula

$$h(x)=\frac{\alpha \gamma }{x^{2}}e^{\frac{\beta }{x}}{\left(e^{\frac{\alpha }{\beta }(e^{\frac{\beta }{x}}-1)}-1\right)}^{-1}.$$ (24)

**Fig 3:**
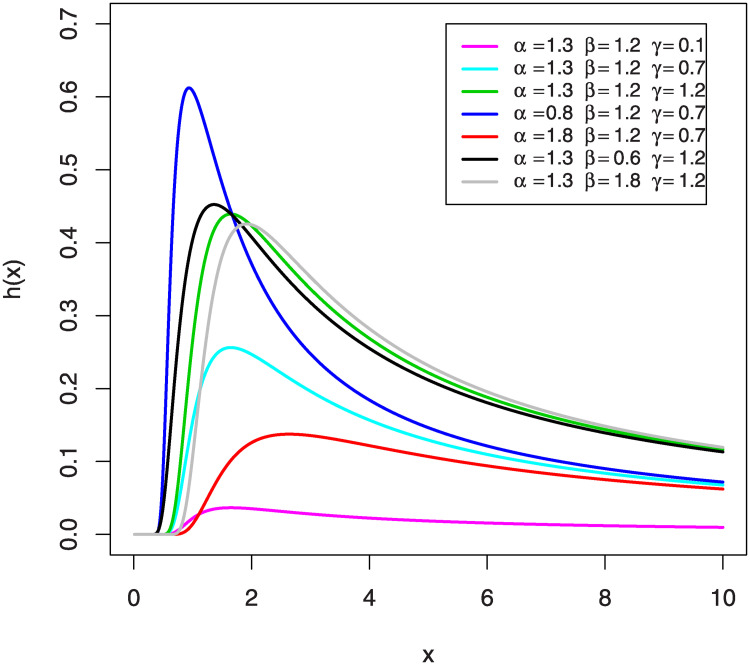
The graphs of the HRF for KuIG.

The graphic behavior of the HRF of KuIG with various choices of *α*, *β* and *γ* is offered in Fig 3.

If *X* ∼ KuIG(**Ω**), then the reversed failure rate function of *X* is

$$r(x)=\frac{\alpha \gamma }{x^{2}}e^{\frac{\beta }{x}}e^{-\frac{\alpha }{\beta }(e^{\frac{\beta }{x}}-1)}{\left(1-e^{-\frac{\alpha }{\beta }(e^{\frac{\beta }{x}}-1)}\right)}^{\gamma -1}{\left\{1-{\left(1-e^{-\frac{\alpha }{\beta }(e^{\frac{\beta }{x}}-1)}\right)}^{\gamma }\right\}}^{-1}.$$ (25)

### 4.2. The mean time to failure

If *X* ∼ KuIG(**Ω**), then the mean time to failure (MTTF) of *X* is found by

$${MTTF=\int_{0}^{\infty }xf(x;\Omega )dx=\mu }^{\left(1\right)}.$$

Substituting from Eq (13), when *r* = 1, we find that

MTTF=∑k=0γ-1∑i=0∞∑j=0i∑m=0∞(γ-1k)(-1)i+j+k+1(k+1)i(1+i)mαi+1β-ij-mγΓ(m)j!m!(i-j)!. (26)

### 4.3. The mean residual lifetime

In life testing situations and reliability theory, the mean residual lifetime (MRL), say *M*_*r*_(*t*), is known as the anticipated remaining lifetime *T* − *t*, provided that the component has been survived until a time *t*. The *M*_*r*_(*t*) is found using the formula

$$M_{r}(t)=E(T-t|T>t).$$

If *T* ∼ KuIG(**Ω**), then the MRL of *T* is

$$\begin{array}{ll} M_{r}(t) & =\frac{1}{R(t)}\int_{t}^{\infty }R(x)dx\\ & =\frac{1}{1-F(t)}[\mu^{\left(1\right)}-\sum_{k=0}^{\gamma }\sum_{i=0}^{\infty }\sum_{j=0}^{i}(\begin{array}{c} \gamma \\ k \end{array})\frac{{\left(-1\right)}^{i+j+k}\alpha^{i}k^{i}\beta^{-i}}{j!(i-j)!}\int_{0}^{t}e^{\frac{(i-j)\beta }{x}}dx]\\ & =\frac{1}{1-F(t)}[\mu^{\left(1\right)}-\sum_{k=0}^{\gamma }\sum_{i=0}^{\infty }\sum_{j=0}^{i}\sum_{m=0}^{\infty }(\begin{array}{c} \gamma \\ k \end{array})\frac{{\left(-1\right)}^{i+j+k}\alpha^{i}k^{i}\beta^{m-i}{\left(i-j\right)}^{m}t^{1-m}}{j!m!(i-j)!(1-m)}]. \end{array}$$ (27)

### 4.4. The mean inactive lifetime

The mean waiting (inactive) lifetime (MWT), say *M*_*w*_(*t*), measures the time elapsed since the component fails with lifetime *T*, provided that it has failed some time before *t*, *t* > 0. It is defined as

$$M_{w}(t)=E(t-T|T\leq t).$$

If *T* ∼ KuIG(**Ω**), then the MWT of *T* is

$$\begin{array}{ll} M_{w}(t) & =\frac{1}{F(t)}\int_{0}^{t}F(x)dx\\ & =\frac{1}{F(t)}[t-\sum_{k=0}^{\gamma }\sum_{i=0}^{\infty }\sum_{j=0}^{i}\sum_{m=0}^{\infty }(\begin{array}{c} \gamma \\ k \end{array})\frac{{\left(-1\right)}^{i+j+k}\alpha^{i}k^{i}\beta^{m-i}{\left(i-j\right)}^{m}t^{1-m}}{j!m!(i-j)!(1-m)}]. \end{array}$$ (28)

## 5. Maximum likelihood estimators for complete data

In this section, we will discuss and study how to use the maximum likelihood approach to appreciate the unknown parameters (*α*, *β*, *γ*) of the KuIG. Suppose that *x*_1_, *x*_2_, …, *x*_*n*_ be a randomly selected sample with size *n* from the KuIG(**Ω**), thus the log-likelihood function *L*(**Ω**) for it is given by

$$L(\Omega )=nln(\alpha \gamma )+\beta \sum_{i=1}^{n}\frac{1}{x_{i}}-2\sum_{i=1}^{n}\text{ln}\left(x_{i}\right)-\frac{\alpha }{\beta }\sum_{i=1}^{n}\left(e^{\frac{\beta }{x_{i}}}-1\right)+(\gamma -1)\sum_{i=1}^{n}\text{ln}\left(1-e^{\frac{-\alpha }{\beta }(e^{\frac{\beta }{x_{i}}}-1)}\right).$$ (29)

By deriving the first partial derivatives of *L*(**Ω**) with regard to *α*, *β* and *γ* and put it equal to zero, the normal equations of *L*(**Ω**) will take the forms

$$\frac{n}{\hat{\gamma }}+\sum_{i=1}^{n}\text{ln}(1-e^{\frac{-\hat{\alpha }}{\hat{\beta }}(e^{\frac{\hat{\beta }}{x_{i}}}-1)})=0,$$ (30)

$$\frac{n}{\hat{\alpha }}-\frac{1}{\hat{\beta }}\sum_{i=1}^{n}\left(e^{\frac{\hat{\beta }}{x_{i}}}-1\right)+\frac{\left(\hat{\gamma }-1\right)}{\hat{\beta }}\sum_{i=1}^{n}\frac{e^{\frac{\hat{\beta }}{x_{i}}}-1}{e^{\frac{\hat{\alpha }}{\hat{\beta }}(e^{\frac{\hat{\beta }}{x_{i}}}-1)}-1}=0$$ (31)

and

$$\sum_{i=1}^{n}\frac{1}{x_{i}}-\frac{\hat{\alpha }}{\hat{\beta }}\sum_{i=1}^{n}{\frac{1}{x_{i}}e}^{\frac{\hat{\beta }}{x_{i}}}+\frac{\hat{\alpha }}{{\hat{\beta }}^{2}}\sum_{i=1}^{n}\left(e^{\frac{\hat{\beta }}{x_{i}}}-1\right)+\frac{\hat{\alpha }(\hat{\gamma }-1)}{{\hat{\beta }}^{2}}\sum_{i=1}^{n}\frac{(\frac{\hat{\beta }}{x_{i}}-1)e^{\frac{\hat{\beta }}{x_{i}}}+1}{e^{\frac{\hat{\alpha }}{\hat{\beta }}(e^{\frac{\hat{\beta }}{x_{i}}}-1)}-1}=0.$$ (32)

Eqs (30), (31) and (32) don’t have an explicit solutions to $\hat{\alpha },\;\hat{\beta }$ and $\hat{\gamma }$. Therefore, we will solve the previous system of equations numerically to obtain the maximum likelihood estimators (MLEs) $\left(\hat{\alpha },\;\hat{\beta },\hat{\gamma }\right)$.

## 6. Maximum likelihood estimators for type-II right censored data

If a life testing experiment stopped over when a limited number of items are observed to be failed, then the remaining items are indicated to be a type-II right censored. The inference associated with this type of data is less efficient than the inference associated with the complete data because some information about the parameters under study will be missed with the type-II right censored data; see [34]. If *x*_(1)_, *x*_(2)_, ……, *x*_(*k*)_, *k* ≤ *n* denote the ordered values of a random sample *x*_1_, *x*_2_, ……, *x*_*n*_ (failure times) and observations terminate after the kth failure occurs, then the likelihood function take the form ([35])

$$l_{cen.}=\frac{n!}{(n-k)!}{\left(R(x_{k})\right)}^{n-k}\prod_{i=1}^{k}f(x_{i}).$$ (33)

If *x*_1_, *x*_2_, …, *x*_*n*_ be a random sample taken from KuIG(**Ω**), then the *L*(**Ω**) of *x*_(1)_, *x*_(2)_, ……, *x*_(*k*)_, *k* ≤ *n* is found by the formula

$$\begin{array}{ll} L(\Omega ) & =kln(\alpha \gamma )+\text{ln}(\frac{n!}{(n-k)!})+(n-k)\text{ln}{\left(1-e^{\frac{-\alpha }{\beta }(e^{\frac{\beta }{x_{k}}}-1)}\right)}^{\gamma }+\beta \overset{k}{\underset{i=1}{\sum }}\frac{1}{x_{i}}-2\overset{k}{\underset{i=1}{\sum }}\text{ln}(x_{i})\\ & -\frac{\alpha }{\beta }\overset{k}{\underset{i=1}{\sum }}(e^{\frac{\beta }{x_{i}}}-1)+(\gamma -1)\overset{k}{\underset{i=1}{\sum }}\text{ln}(1-e^{\frac{-\alpha }{\beta }(e^{\frac{\beta }{x_{i}}}-1)}). \end{array}$$ (34)

The first partial derivatives of *L*(**Ω**) are obtained by differentiating Eq (34) for *α*, *β* and *γ* as

$$\frac{\partial L}{\partial \gamma }=\frac{k}{\gamma }+\sum_{i=1}^{k}\text{ln}(1-e^{\frac{-\alpha }{\beta }(e^{\frac{\beta }{x_{i}}}-1)}),$$ (35)

$$\frac{\partial L}{\partial \alpha }=\frac{k}{\alpha }+\frac{\gamma (n-k)(e^{\frac{\beta }{x_{k}}}-1)}{\beta (e^{\frac{\alpha }{\beta }(e^{\frac{\beta }{x_{k}}}-1)}-1)}-\frac{1}{\beta }\sum_{i=1}^{k}\left(e^{\frac{\beta }{x_{i}}}-1\right)+\frac{\left(\gamma -1\right)}{\beta }\sum_{i=1}^{k}\frac{e^{\frac{\beta }{x_{i}}}-1}{e^{\frac{\alpha }{\beta }(e^{\frac{\beta }{x_{i}}}-1)}-1}$$ (36)

and

$$\frac{\partial L}{\partial \beta }=\frac{\alpha \gamma (n-k)((\frac{\beta }{x_{k}}-1)e^{\frac{\beta }{x_{k}}}+1)}{\beta^{2}(e^{\frac{\alpha }{\beta }(e^{\frac{\beta }{x_{k}}}-1)}-1)}+\overset{k}{\underset{i=1}{\sum }}\frac{1}{x_{i}}-\frac{\alpha }{\beta }\overset{k}{\underset{i=1}{\sum }}\frac{1}{x_{i}}e^{\frac{\beta }{x_{i}}}+\frac{\alpha }{\beta^{2}}\overset{k}{\underset{i=1}{\sum }}(e^{\frac{\beta }{x_{i}}}-1)+\frac{\alpha (\gamma -1)}{\beta^{2}}\overset{k}{\underset{i=1}{\sum }}\frac{(\frac{\beta }{x_{i}}-1)e^{\frac{\beta }{x_{i}}}+1}{e^{\frac{\alpha }{\beta }(e^{\frac{\beta }{x_{i}}}-1)}-1}.$$ (37)

Equating Eqs (35), (36) and (37) to zero, we will get the normal equations of *L* which don’t have an explicit solutions to $\hat{\alpha },\;\hat{\beta }$, and $\hat{\gamma }$ and must be solved numerically to find the maximum likelihood estimators (MLEs) $\left(\hat{\alpha },\;\hat{\beta },\hat{\gamma }\right)$.

## 7. Maximum likelihood estimators for upper record data

The study of record values has extensive applications to real world situations such as sporting events, meteorological and seismological sciences and life testing studies. The upper record value is that one which is larger than all watched values so far. Suppose that *X* = {*X*_*U*(1)_, *X*_*U*(2)_, …, *X*_*U*(*n*)_} is an upper record values taken from a random sample *x*_1_, *x*_2_, …, *x*_*n*_ that follow the KuIG, then the likelihood function of *X* can be expressed by [36]

$$l_{reco.}=f(x_{U(n)};\Omega )\prod_{i=1}^{n-1}\frac{f(x_{U(i)},\;\Omega )}{R(x_{U(i)},\;\Omega )},\;0\leq x_{U(1)}<x_{U(2)}<\cdots <x_{U(n)}<\infty .$$ (38)

From Eqs (7) and (8), we can obtain the *L*(**Ω**) as follows

$$\begin{array}{ll} L(\Omega ) & =nln(\alpha \gamma )-\text{ln}(x_{U(n)}^{2})+\frac{\beta }{x_{U(n)}}-\frac{\alpha }{\beta }(e^{\frac{\beta }{x_{U(n)}}}-1)+(\gamma -1)\text{ln}(1-e^{\frac{-\alpha }{\beta }(e^{\frac{\beta }{x_{U(n)}}}-1)})\\ & -\overset{n-1}{\underset{i=1}{\sum }}\text{ln}(x_{U(i)}^{2})+\beta \overset{n-1}{\underset{i=1}{\sum }}\frac{1}{x_{U(i)}}-\overset{n-1}{\underset{i=1}{\sum }}\text{ln}(e^{\frac{\alpha }{\beta }(e^{\frac{\beta }{x_{U(i)}}}-1)}-1). \end{array}$$ (39)

Differentiating Eq (39), we will obtain the first partial derivatives of *L*(**Ω**) with regard to *α*, *β*, and *γ* as

$$\frac{\partial L}{\partial \gamma }=\frac{n}{\gamma }+ln(1-e^{\frac{-\alpha }{\beta }(e^{\frac{\beta }{x_{U(n)}}}-1)}),$$ (40)

$$\frac{\partial L}{\partial \alpha }=\frac{n}{\alpha }-\frac{1}{\beta }(e^{\frac{\beta }{x_{U(n)}}}-1)+\frac{\left(\gamma -1)(e^{\frac{\beta }{x_{U(n)}}}-1\right)}{\beta (e^{\frac{\alpha }{\beta }(e^{\frac{\beta }{x_{U(n)}}}-1)}-1)}-\frac{1}{\beta }\sum_{i=1}^{n-1}\frac{e^{\frac{\beta }{x_{U(i)}}}-1}{1-e^{\frac{-\alpha }{\beta }(e^{\frac{\beta }{x_{U(i)}}}-1)}}$$ (41)

and

$$\frac{\partial L}{\partial \beta }=\frac{1}{x_{U(n)}}-\frac{\alpha }{\beta^{2}}((\frac{\beta }{x_{U(n)}}-1)e^{\frac{\beta }{x_{U(n)}}}+1)(1-\frac{\gamma -1}{e^{\frac{\alpha }{\beta }(e^{\frac{\beta }{x_{U(n)}}}-1)}-1})+\sum_{i=1}^{n-1}\frac{1}{x_{U(i)}}-\frac{\alpha }{\beta^{2}}\sum_{i=1}^{n-1}\frac{(\frac{\beta }{x_{U(i)}}-1)e^{\frac{\beta }{x_{U(i)}}}+1}{1-e^{\frac{-\alpha }{\beta }(e^{\frac{\beta }{x_{U(i)}}}-1)}}$$ (42)

By equal Eqs (40), (41) and (42) to zero, we will get the normal equations of *L* which don’t have an explicit solutions to $\hat{\alpha },\;\hat{\beta }$, and $\hat{\gamma }$ and must be solved numerically to find the MLEs $\left(\hat{\alpha },\;\hat{\beta },\hat{\gamma }\right)$.

## 8. Simulation results

**Fig 4:**
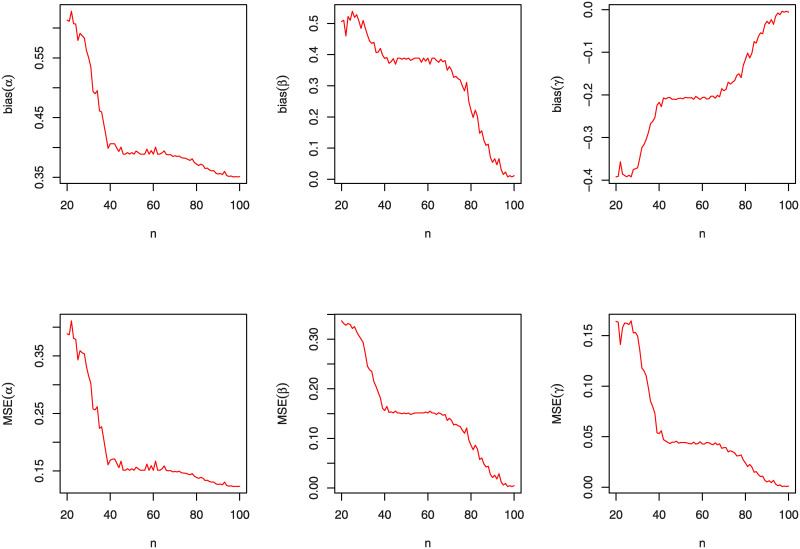
The bias estimates and MSEs of the KuIG for various values of *n* when (*α*, *β*, *γ*) = (0.1, 3.1, 3.5).

**Fig 5:**
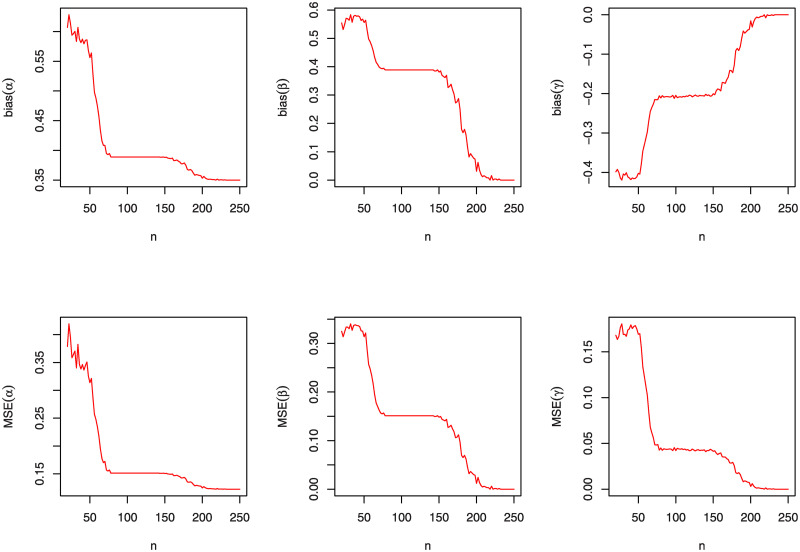
The bias estimates and MSEs of the KuIG for various values of *n* when (*α*, *β*, *γ*) = (0.3, 2.1, 3.5).

**Fig 6:**
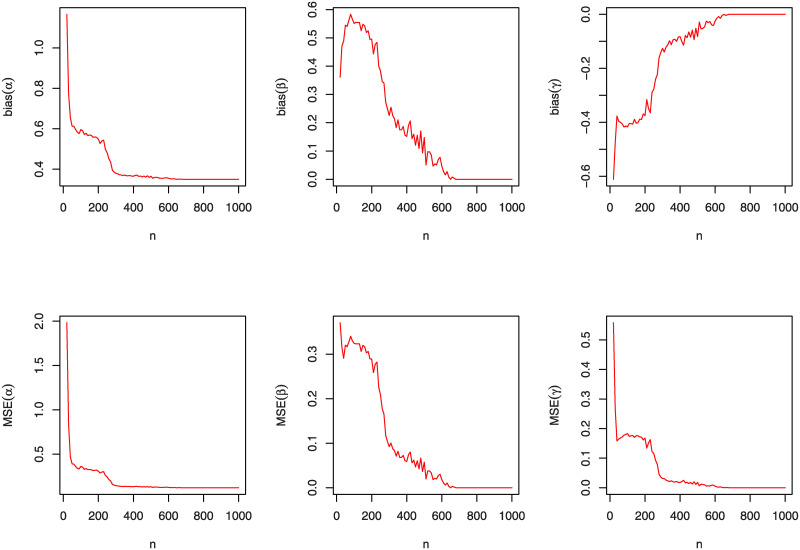
The bias estimates and MSEs of the KuIG for various values of *n* when (*α*, *β*, *γ*) = (0.7, 2.1, 3.5).

A simulation study will be accomplished, in this section, to appreciate the performance of the MLEs $\left(\hat{\alpha },\;\hat{\beta },\hat{\gamma }\right)$ by using the bias estimates and the mean squared errors (MSEs); see [37]. This simulation is carried out by using "R" language. We examine the behavior of the MLEs for varied values of *n*, *α*, *β* and *γ*. Furthermore, the graphical comparison of these three parameters according to the bias estimates and MSEs for the KuIG distribution is displayed in Figs 4, 5 and 6 which demonstrates that both bias estimates and MSEs are decreased as *n* increases. That is, estimating the parameters of the KuIG by using the MLE technique implements quite well.

## 9. Data analysis

We will analyze here five real data sets, three of them are complete, one is type-II right censored and one is upper record data to clarify that the KuIG distribution is a good lifetime model, compared with many known models like inverse flexible Weibull (IFW), exponentiated inverse flexible Weibull (EIFW), inverse Weibull (IW), inverse exponential (IE), inverse Rayleigh (IR), A distribution (A), and inverse Gompertz (IG) distributions. The MLE method will be used to compare the goodness-of-fit of the KuIG with these distributions. All mentioned distributions will be fitted in each data set according to some different criteria, namely, the Kolmogorov Smirnov test statistic (KS) with its corresponding P-values. Also, the log-likelihood values (L), Akaike information criterion (AIC), correct Akaike information criterion (CAIC), Hannan-Quinn information criterion (HQIC), Bayesian information criterion (BIC), Cramér-von Mises (W*) statistic and Anderson-Darling (A*) statistic will be found; see [38–43].

### 9.1. Complete data set I

The first data mentioned by [44] and represents the strength of glass for aircraft window (see, A1 in S1 Appendix). The MLEs, KS and P-values are given in Table 1 for all eight studied models. It is obvious that the KuIG has the smallest KS value and the largest P-value through whole models elaborated. This emphasizes that the KuIG fits the first data better than IE, IR, IW, A, IFW, EIFW and IG models. On the other hand, for the eight mentioned models, the KuIG has the smallest values of–L, AIC, CAIC, HQIC, BIC, W* and A*. This confirms that the KuIG appears to be a very competitive model to data I better than the other seven models.

**Table 1:** The MLEs, KS, P-values, –L, AIC, CAIC, HQIC, BIC, A* and W* values for data I.

Statistics	Models							
	IFW	EIFW	IW	IR	IE	A	IG	KuIG
$\hat{\alpha }$	61.167	2.376	4461827	810.504	29.215	125.662	1.249	79.042
$\hat{\beta }$	0.0859	0.164	4.655	––	––	––	119.762	18.694
$\hat{\gamma }$	––	81.512	––	––	––	––	––	26.554
**KS**	0.146	0.136	0.146	0.325	0.477	0.162	0.139	0.124
**P-value**	0.479	0.567	0.482	0.002	6.15×10^−7^	0.354	0.538	0.681
–**L**	104.963	104.141	105.323	118.201	137.262	107.950	107.884	103.988
**AIC**	213.927	214.282	214.647	238.401	276.523	217.901	219.768	213.976
**CAIC**	214.355	215.171	215.075	238.539	276.661	218.039	220.196	214.865
**BIC**	216.795	218.584	217.515	239.835	277.957	219.335	222.636	218.278
**HQIC**	214.862	215.684	215.582	238.869	276.990	218.368	220.702	215.379
W*	0.078	0.0742	0.083	0.075	0.074	0.122	0.118	0.074
A*	0.467	0.397	0.503	0.403	0.392	0.804	0.778	0.394

**Fig 7:**
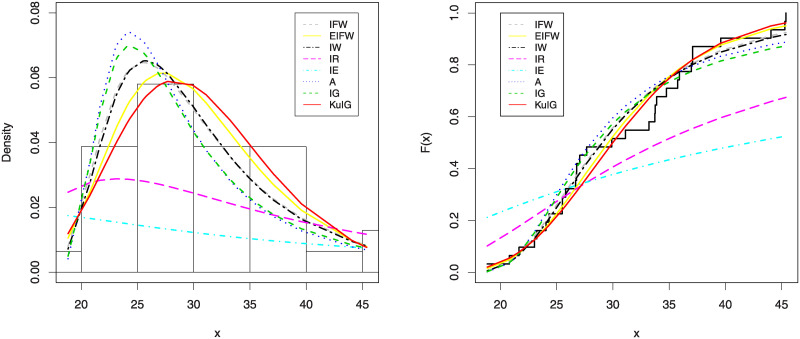
The estimated PDFs (left panel) and the estimated CDFs (right panel) of data I.

**Fig 8:**
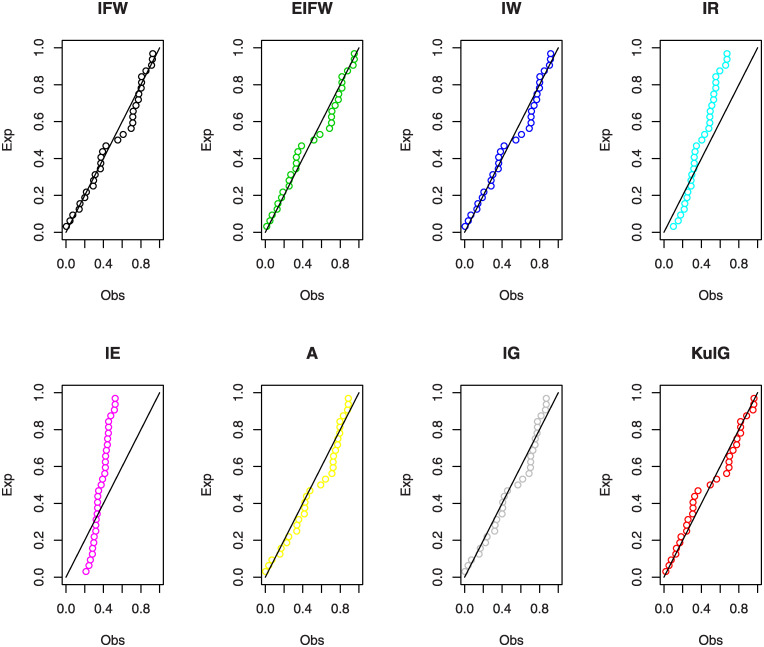
The P-P plots for data I.

In Figs 7 and 8, we show the estimated PDFs, estimated CDFs and P-P plots of all tested distributions using the estimators obtained in Table 1. From these figures, it is noticed that the KuIG fits the first data better than the other seven models. The likelihood ratio test (LRT) can be used here to test if the fit by KuIG model is statistically superior to the fit by A and IG models for data set I. Table 2 gives the values of the LRT, degree of freedom (**d.f**) and its P-values for data I. Based on the P-values, we will reject the null hypothesis (*H*_0_) at a level of significance ***α*** = 0.05.

**Table 2:** The LRT, degree of freedom and P-value for data I.

Models	Null Hypothesis (*H*_0_)	Λ	*D*.*F*	*P*-value
**A**	*α* = *γ* = 1 or *x*_1_, *x*_2_, …, *x*_*n*_ ~ *A*(*β*)	7.925	2	0.019
**IG**	*γ* = 1 or *x*_1_, *x*_2_, …, *x*_*n*_ ~ *IG*(*β*)	7.792	1	0.005

**Fig 9:**
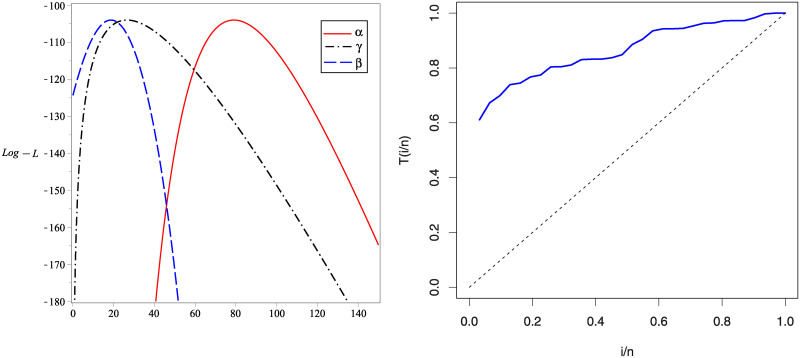
The profile of *L*(Ω) for data set I (left panel) and the TTT plot (right panel).

The profile of *L*(**Ω**) for the parameters of KuIG in case of the first real set of data is given in Fig 9 which confirms that only one solution is existed for the likelihood equations. The total test time (TTT) plot which specifies some qualitative readings about the failure rate shape is also given in Fig 9.

### 9.2. Complete data set II

**Fig 10:**
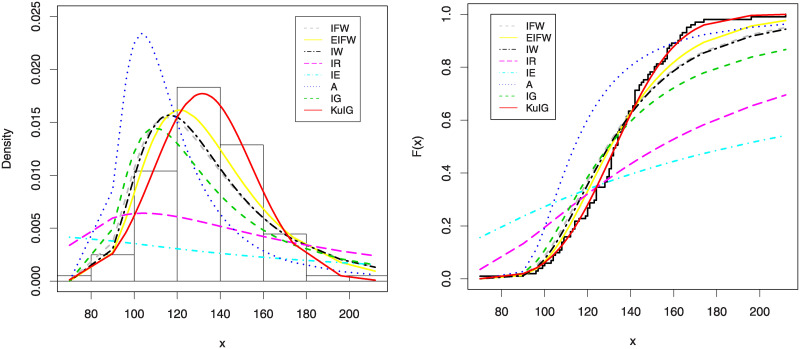
The estimated PDFs (left panel) and the estimated CDFs (right panel) of data II.

**Fig 11:**
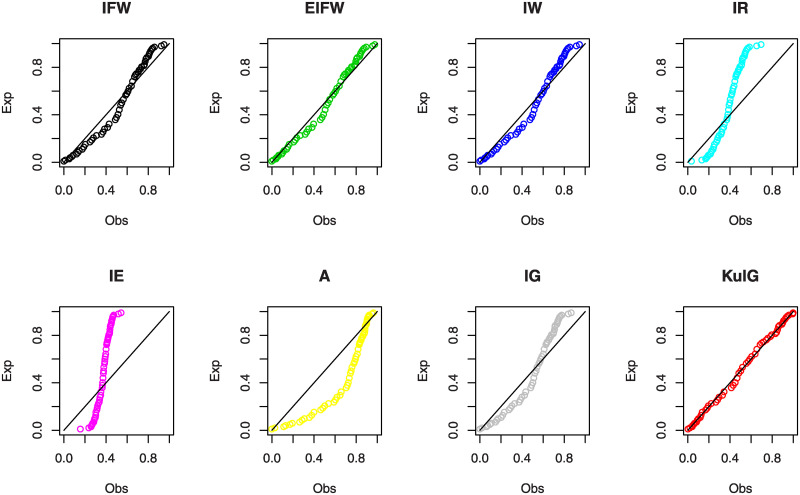
The P-P plots of data II.

**Fig 12:**
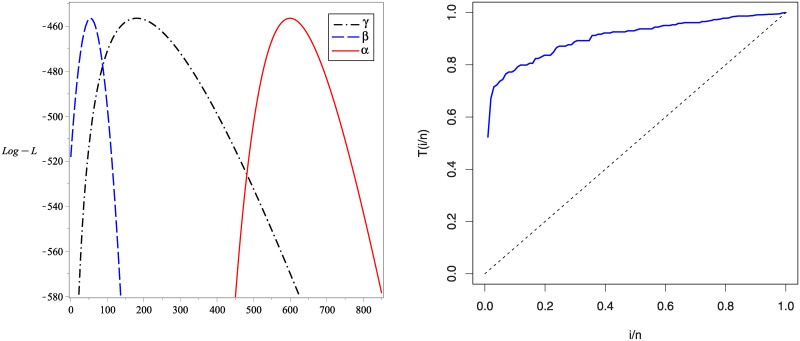
The profile of *L*(Ω) for data II (left panel) and the TTT plot (right panel).

The second data introduced by [45] represents the tiredness lifetime of 101 6061-T6 aluminum coupons (see, A2 in S1 Appendix). Table 3 presents the MLEs, KS and the P-values for all compared distributions. Also, the values of–L, AIC, CAIC, HQIC, BIC, A* and W* are determined for these distributions. From Table 3, we find that the KuIG is the best model between all studied distributions. Figs 10 and 11 shows the estimated PDFs, estimated CDFs and P-P plots of the tested distributions using the estimators obtained in Table 3. These figures confirm that the KuIG fits data II better than all seven tested models. Fig 12 gives the profile of the *L*(**Ω**) of the parameters *α*, *β* and *γ* and the TTT-plot for the second real set of data. From the profile of the *L*(**Ω**), we conclude that one and only solution is existed for the likelihood equations. Table 4 presents the values of LRT, d.f with the P-values for data set II. We deduce that the null hypotheses (*H*_0_) are rejected at ***α*** = 0.05.

**Table 3:** The MLEs, KS, P-values, –L, AIC, CAIC, HQIC, BIC, A* and W* values for data II.

Statistics	Models							
	IFW	EIFW	IW	IR	IE	A	IG	KuIG
$\hat{\alpha }$	295.466	78.792	3.28×10^10^	16361.23	129.933	**705.55**	7.435	599.595
$\hat{\beta }$	0.0206	0.0386	5.051	––	––	––	501.775	53.955
$\hat{\gamma }$	––	58.724	––	––	––	––	––	180.479
**KS**	0.139	0.113	0.133	0.403	0.506	0.366	0.206	0.067
**P-value**	0.039	0.153	0.055	1.21×10^−14^	0.0	3.77×10^−12^	0.00038	0.761
–**L**	476.101	465.265	475.186	530.197	595.547	517.597	494.448	456.431
**AIC**	956.202	936.531	954.372	1062.393	1193.094	1037.194	992.896	918.862
**CAIC**	956.325	936.778	954.494	1062.434	1193.135	1037.234	993.018	919.109
**BIC**	961.432	944.376	959.602	1065.009	1195.709	1039.809	998.126	926.707
**HQIC**	958.319	939.707	956.489	1063.452	1194.153	1038.253	995.013	922.038
W*	0.437	0.238	0.432	0.172	0.121	1.204	0.803	0.056
A*	2.548	1.349	2.493	0.975	0.689	7.025	4.707	0.360

**Table 4:** The LRT, degree of freedom and P-value of data II.

Models	Null Hypothesis (*H*_0_)	Λ	*D*.*F*	*P*-value
**A**	*α* = *γ* = 1 or *x*_1_, *x*_2_, …, *x*_*n*_ ~ *A*(*β*)	122.332	2	0
**IG**	*γ* = 1 or *x*_1_, *x*_2_, …, *x*_*n*_ ~ *IG*(*β*)	76.034	1	0

### 9.3. Complete data set III

**Fig 13:**
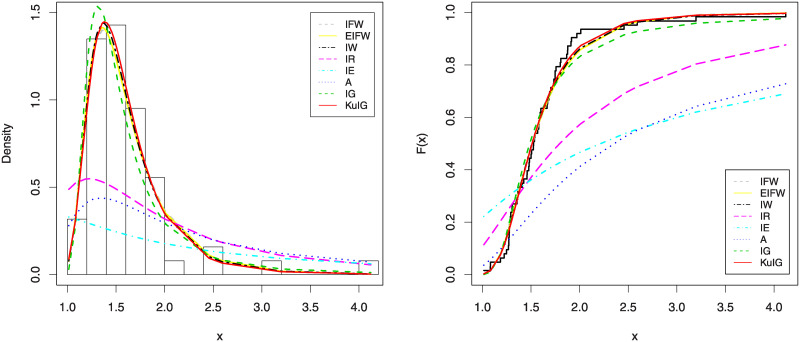
The estimated PDFs (left panel) and the estimated CDFs (right panel) for data III.

**Fig 14:**
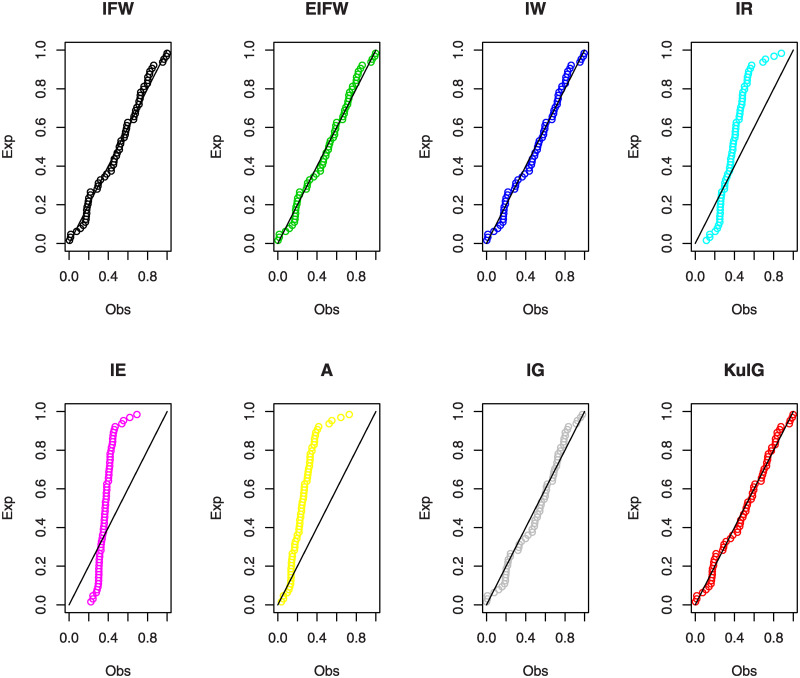
The P-P plots of data III.

**Fig 15:**
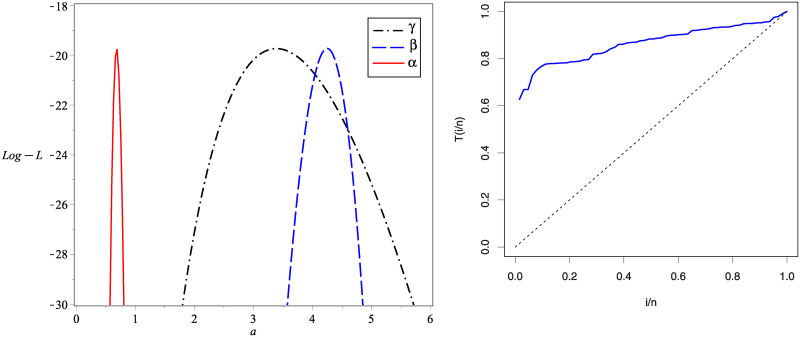
The profile of *L*(Ω) for data set III (left panel) and the TTT plot (right panel).

The third complete data set symbolizes the simulated strengths of glass fibers presented by [46] (see, A3 in S1 Appendix). In Table 5, we present the MLEs, KS and the P-values for all tested distributions. Moreover, the values of–L, AIC, CAIC, HQIC, BIC, A* and W* are determined for these distributions. From Table 5, we find that the KuIG is the best model between all studied distributions. The estimated PDFs, estimated CDFs with the P-P plots of the tested distributions using the estimators obtained in Table 5 are presented in Figs 13 and 14. These figures confirm that the KuIG fits data set III better than all seven tested models. Fig 15 shows the profile of the *L*(**Ω**) of the parameters *α*, *β* and *γ* and the TTT-plot. It is appeared, from the profile of the *L*(**Ω**), that one and only solution exists for the likelihood equations. Table 6 describes the values of LRT, d.f with the P-values for data III. According to the P-values, the null hypothesis (*H*_0_) will be refused at ***α*** = 0.05.

**Table 5:** The MLEs, KS, P-values, –L, AIC, CAIC, HQIC, BIC, A* and W* values of data III.

Statistics	Models							
	IFW	EIFW	IW	IR	IE	A	IG	KuIG
$\hat{\alpha }$	3.732	4.169	6.498	2.233	1.526	2.111	0.032	0.684
$\hat{\beta }$	1.869	1.666	5.438	––	––	––	7.583	4.223
$\hat{\gamma }$	––	0.544	––	––	––	––	––	3.452
**KS**	0.082	0.084	0.077	0.360	0.468	0.521	0.101	0.068
**P-value**	0.756	0.739	0.819	7.762×10^−8^	3.537×10^−13^	4.44×10^−16^	0.508	0.917
–**L**	20.618	20.593	20.064	53.381	92.805	63.322	22.809	19.719
AIC	45.237	47.186	44.128	108.762	187.609	128.645	49.617	45.439
CAIC	45.437	47.593	44.328	108.827	187.675	128.710	49.817	45.847
BIC	49.523	53.615	48.414	110.905	189.753	130.788	53.903	51.869
HQIC	46.923	49.715	45.814	109.604	188.452	129.487	51.303	47.969
W*	0.079	0.081	0.071	0.087	0.126	0.0629	0.138	0.0610
A*	0.610	0.616	0.533	0.709	0.982	0.514	0.928	0.471

**Table 6:** The LRT, degree of freedom and P-value for data set III.

Models	Null Hypothesis (*H*_0_)	Λ	*D*.*F*	*P*-value
**A**	*α* = *γ* = 1 or *x*_1_, *x*_2_, …, *x*_*n*_ ~ *A*(*β*)	87.204	2	0
**IG**	*γ* = 1 or *x*_1_, *x*_2_, …, *x*_*n*_ ~ *IG*(*β*)	6.177	1	0.013

### 9.4. Data set IV (Type-II right censored data)

The censored data analyzed here was introduced by [47] which represents the fatigue life for 10 bearings of a specific type in hours and a sample of size k = 8 from this data is taken (see, A4 in S1 Appendix). Table 7 shows the MLEs, –L, and KS with the P-values for the A, IG and KuIG distributions. It is clear that, the KuIG has the lowest -L and KS value and the highest P-value and this emphasizes that KuIG fits the studied data here better than A and IG.

**Table 7:** The MLEs, K-S, P-values and –L values for type-II right censored data.

Models	MLEs	-L	KS	P-value
A	$\hat{\alpha }=1295.5$	29.224	0.367	0.135
IG	$\hat{\alpha }=2.211,\;\hat{\beta }=1.144$	24.357	0.349	0.175
**KuIG**	$\hat{\alpha }=1.560,\;\hat{\beta }=1201.6,\;\hat{\gamma }=0.909$	22.971	0.159	0.961

### 9.5. Data set V (Upper record data)

The record data analyzed here are introduced by [48] which contains n = 11 lifetimes to breakdown of an electric isolating fluid exposed to thirty kilovolt (see, A5 in S1 Appendix). From this data, it is found that the upper record values are 2.836, 3.120, 5.169 and 5.272. Table 8 summarizes the MLEs, –L, and KS with the P-values for A, IG and KuIG distributions. It is clear that, the KuIG has the lowermost -L and KS value and the uppermost P-value and this emphasizes that the KuIG fits this type of data better than the A and IG models.

**Table 8:** The MLEs, –L, KS, and P-values for upper record data.

Models	MLEs	-L	KS	P-value
A	$\hat{\alpha }=7.136$	7.592	0.687	0.046
IG	$\hat{\alpha }=2.82\times 10^{-3},\;\hat{\beta }=26.399$	3.25	0.513	0.285
**KuIG**	$\hat{\alpha }=0.204,\;\hat{\beta }=13.606,\;\hat{\gamma }=2.235$	2.993	0.479	0.317

## 10. Conclusions

In this article, we proposed a new model named KuIG which is considered as an extension and generalization to inverse Gompertz and A distributions. The studied model is characterized by an upside down bathtub-shaped curve hazard rate function depending upon the shape parameters. Also, the KuIG is appropriate for testing the goodness of fit of some special sub-models, such as the IG and A distributions. Some statistical and mathematical properties including the quantiles, median, mode, moments, PWMs, entropies, skewness and kurtosis of KuIG are derived and discussed. Also, some basic functions used in reliability theory are obtained such as the reliability function, HRF, reversed HRF, MTTF, MRL and MWT. The parameters of KuIG are appreciated using the MLE in case of complete, type-II right censored and upper record data. A simulation is done to determine the performance of MLEs according to biases and MSEs. Three complete data sets are analyzed using the KuIG and it is compared with seven other life time distributions. Also, we analyzed a type-II right censored and upper record data and it is compared with the IG and A distributions. It is found that the KuIG has more flexibility for fitting the various data in engineering applications than the mentioned models. Future works include (i) bivariate extension of the KuIG, (ii) KuIG-G family of distributions, (iii) different estimation methods of the KuIG and (iv) discrete case of the KuIG.

## Supporting information

S1 Appendix (DOCX)
