# Supplementary material for: Kumaraswamy inverse Gompertz distribution: Properties and engineering applications to complete, type-II right censored and upper record data
Source: PLoS One. 2020 Dec 3;15(12):e0241970. doi: 10.1371/journal.pone.0241970 (PMC7714165; doi:10.1371/journal.pone.0241970)
Supplement: S1 Appendix — (DOCX) [file pone.0241970.s001.docx]

**S1 Appendix**

**A1. Data I**

| 18.83 | 20.8 | 21.657 | 23.03 | 23.23 | 24.05 | 24.321 | 25.5 | 25.52 | 25.8 | 26.69 |
| --- | --- | --- | --- | --- | --- | --- | --- | --- | --- | --- |
| 26.77 | 26.78 | 27.05 | 27.67 | 29.9 | 31.11 | 33.2 | 33.73 | 33.76 | 33.89 | 34.76 |
| 35.75 | 35.91 | 36.98 | 37.08 | 37.09 | 39.58 | 44.045 | 45.29 | 45.381 |  | |

**A2. Data II**

| 70 | 90 | 96 | 97 | 99 | 100 | 103 | 104 | 104 | 105 | 107 | 108 | 108 | 108 | 109 | 109 | 112 |
| --- | --- | --- | --- | --- | --- | --- | --- | --- | --- | --- | --- | --- | --- | --- | --- | --- |
| 112 | 113 | 114 | 114 | 114 | 116 | 119 | 120 | 120 | 120 | 121 | 121 | 123 | 124 | 124 | 124 | 124 |
| 124 | 128 | 128 | 129 | 129 | 130 | 130 | 130 | 131 | 131 | 131 | 131 | 131 | 132 | 132 | 132 | 133 |
| 134 | 134 | 134 | 134 | 134 | 136 | 136 | 137 | 138 | 138 | 138 | 139 | 139 | 141 | 141 | 142 | 142 |
| 142 | 142 | 142 | 142 | 144 | 144 | 145 | 146 | 148 | 148 | 149 | 151 | 151 | 152 | 155 | 156 | 157 |
| 157 | 157 | 157 | 158 | 159 | 162 | 163 | 163 | 164 | 166 | 166 | 168 | 170 | 174 | 196 | 212 |  |

**A3. Data III**

| 1.014 | 1.081 | 1.082 | 1.185 | 1.223 | 1.248 | 1.267 | 1.271 | 1.272 | 1.275 | 1.276 | 1.278 | 1.286 |
| --- | --- | --- | --- | --- | --- | --- | --- | --- | --- | --- | --- | --- |
| 1.288 | 1.292 | 1.304 | 1.306 | 1.355 | 1.361 | 1.364 | 1.379 | 1.409 | 1.426 | 1.459 | 1.460 | 1.476 |
| 1.481 | 1.484 | 1.501 | 1.506 | 1.524 | 1.526 | 1.535 | 1.541 | 1.568 | 1.579 | 1.581 | 1.591 | 1.593 |
| 1.602 | 1.666 | 1.670 | 1.684 | 1.691 | 1.704 | 1.731 | 1.735 | 1.747 | 1.748 | 1.757 | 1.800 | 1.806 |
| 1.867 | 1.876 | 1.878 | 1.910 | 1.916 | 1.972 | 2.012 | 2.456 | 2.592 | 3.197 | 4.121 |  |  |

**A4. Data IV**

| 152.7 | 172 | 172.5 | 173.5 | 193 | 204.7 | 216.5 | 234.9 | 262.6 | 422.6 |
| --- | --- | --- | --- | --- | --- | --- | --- | --- | --- |

**A5. Data V**

| 2.836 | 3.120 | 3.045 | 5.169 | 4.934 | 4.970 | 3.018 | 3.770 | 5.272 | 3.856 | 2.046 |
| --- | --- | --- | --- | --- | --- | --- | --- | --- | --- | --- |
